# Association between family caregivers' primary care experience when they report as patients and their stress related to caregiving: A pilot cross‐sectional study

**DOI:** 10.1002/jgf2.631

**Published:** 2023-06-13

**Authors:** Gen Nakayama, Shoichi Masumoto, Junji Haruta, Tetsuhiro Maeno

**Affiliations:** ^1^ Department of Primary Care and Medical Education Institute of Medicine, University of Tsukuba Tsukuba Japan; ^2^ Department of Family Medicine, General Practice and Community Health Institute of Medicine, University of Tsukuba Tsukuba Japan; ^3^ Department of General Medicine Tsukuba Central Hospital Ushiku Japan; ^4^ Medical Education Center, School of Medicine Keio University Tokyo Japan; ^5^ Center for General Medicine Education, School of Medicine Keio University Tokyo Japan

**Keywords:** caregivers, caregiver burden, caregiver stress, health care quality assurance, person‐centered care, primary health care

## Abstract

**Background:**

Few studies have examined whether family caregivers' own primary care providers can affect caregiving‐specific well‐being, such as caregiver stress. In this pilot study, we explored whether primary care experiences when family caregivers report as patients were associated with the stress of caregiving.

**Methods:**

We used cross‐sectional data from a survey conducted in Japan between November and December 2020. We recruited family caregivers aged 40–74 years who were caring for community‐dwelling adults with chronic conditions. We assessed primary care experience using the Japanese version of the Primary Care Assessment Tool Short Form (JPCAT‐SF) and caregiver stress using the Japanese short version of the Zarit Caregiver Burden Interview.

**Results:**

In total, 406 family caregivers were included in the analysis. The mean JPCAT‐SF total score was 42.1 out of 100 points. The proportion of caregivers who had higher caregiver stress was 48.8%. After adjusting for possible confounders, the JPCAT‐SF score was found to be significantly associated with caregiver stress (lower stress = 0 vs. higher stress = 1; adjusted prevalence ratio per 1 SD increase in JPCAT‐SF score = 0.89; 95% CI 0.80–0.98). Among the subscales of the JPCAT‐SF, longitudinality, and comprehensiveness (services available) were associated with caregiver stress.

**Conclusions:**

Better primary care experiences when family caregivers reported as patients were associated with lower caregiver stress. Longitudinality, which includes focusing attention on the individual as a whole person, and comprehensiveness in the context of building provider‐patient relationships that make consultation easier when needed, were associated with lower stress.

## Graphical abstract

In caring for family caregivers, the caregivers’ own primary care providers can be support resources; however, studies investigating whether those providers can affect caregiving‐specific well‐being, such as caregiver stress, are scarce. We explored whether primary care experiences when family caregivers report as patients were associated with their stress related to caregiving. We found that better primary care experiences were associated with lower caregiver stress.

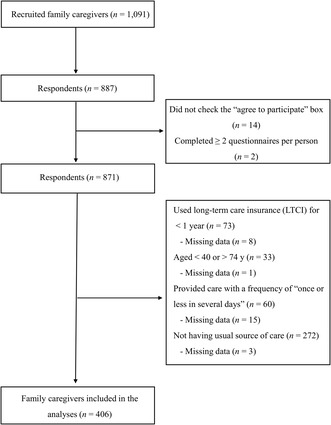

## 1 INTRODUCTION

With a rapidly aging world population and increasing incidence of chronic illness, older people rely increasingly on family members to support their daily activities, extending the roles and needs of family caregivers.^1^ Family caregivers, also known as informal caregivers, refer to unpaid family members or important individuals who offer practical assistance to an ill or aged relative that cannot carry out essential tasks.^1^ Family caregivers have reported more problems with psychological and physical health than non‐caregivers.^2^ In a cohort study, family caregivers who cared for a disabled spouse and reported mental or emotional stress had a higher mortality rate than non‐caregivers or caregivers who did not report the stress of caregiving.^3^ Thus, it is ideal for health care providers to consider the needs, wishes, and stress levels of family caregivers as well as the care recipients; this is especially expected in primary care.^4^

Previous research has examined whether the support provided by care recipients' primary care providers can affect caregiving‐specific well‐being, such as caregiver stress,^5^, ^6^, ^7^ but few have investigated the impact of caregivers' own primary care providers on their stress. Care recipients and caregivers are not always registered in the same general practice, even in countries with established gatekeeping systems, such as the United Kingdom.^8^ In the context of care recipients' primary care providers, a prospective study conducted in the United States showed that family caregivers who reported greater support from care recipients' providers subsequently perceived caregiving as a more positive experience.^5^ On the other hand, in the context of family caregivers' own primary care providers, a German study observed that 77% of caregivers who went to a primary care physician discussed their care situation.^9^ Another German study showed that family caregivers perceived the support provided by their own physicians favorably in terms of psychosocial support.^10^ Hence, we expected that family caregivers who received higher quality primary care as a patient may receive more psychosocial attention, including the context of their caregiving responsibilities, and consequently experience less stress related to caregiving. However, to our knowledge, no study worldwide has specifically examined whether the support offered by those providers can affect caregiver stress.

Patient experience is recognized as a valid quality indicator of person‐centeredness.^11^, ^12^ Person‐centeredness, also known as patient‐centeredness, means providing care that is respectful of and responsive to individual preferences, needs, and values.^13^ Measuring patient experience is seen as a useful tool for assessing the quality of health care,^11^ and this is particularly noticeable in primary care.^14^

In this pilot study, we aimed to explore whether family caregivers' primary care experience when reporting as a patient was associated with their stress related to caregiving, using data from a cross‐sectional survey.

## 2 METHODS

### 2.1 Design, setting, participants, and procedures

The cross‐sectional data for this study were drawn from a cross‐sectional survey with several planned analyses in Ibaraki Prefecture, situated in the northeastern part of the Greater Tokyo Area, between November and December 2020. The survey recruited family caregivers caring for community‐dwelling adults with chronic illnesses. The care recipients were using Japan's Long‐Term Care Insurance (LTCI) system. Family caregivers were recruited consecutively through “care managers,” who support the caregivers and their care recipients under LTCI. Data were collected using a self‐administered questionnaire. Family caregivers provided informed consent via the questionnaires and returned the questionnaires by mail to our university office.

#### 2.1.1 Inclusion criteria

Study participants were eligible for the survey if they were aged between 40 and 74 years, and were caring for a care recipient who had been using LTCI for ≥1 year. The reasons for setting these inclusion criteria are detailed in a previous paper.^15^

#### 2.1.2 Exclusion criteria

Family caregivers were excluded from the study under two conditions: if they answered ≥2 questionnaires per person because a family caregiver caring for two or more people was instructed to only answer questions related to the care of the most dependent person; and if provided care with a frequency of “once or less in several days,”^16^ because those who provide less frequent care were considered to be more heterogeneous. These criteria are the same as studies from the survey.^15^, ^17^ Family caregivers without a usual source of care were also excluded from the study, as required by the scale assessing primary care experience (see below).

### 2.2 Measures

#### 2.2.1 Outcome variable: Family caregivers' stress related to caregiving

We assessed family caregivers' stress related to caregiving using the Japanese short version of the Zarit Caregiver Burden Interview (J‐ZBI_8).^18^ The Zarit Caregiver Burden Interview,^19^, ^20^ which was the original instrument for J‐ZBI_8 is one of the most commonly used measures of caregiver stress.^21^, ^22^ The concept of caregiver stress is commonly used in the literature to describe the burden and strain that family caregivers experience related to caregiving.^21^, ^22^

J‐ZBI_8 consists of eight questions on a 5‐point Likert scale, with two factors: personal strain and role strain. Personal strain refers to how the caregiving experience is personally stressful,^23^ including items such as “Do you feel angry when you are around this person?” and “Do you wish you could just leave the care of your relative to someone else?” Role strain refers to the stress because of the conflicting roles the caregiver has to manage in life,^23^ including, for example, the item “Do you feel that your social life has suffered because you are caring for your relative?” Personal strain has five items, whereas role strain has three; therefore, the former accounts for a larger proportion of the scale's total score. The total score ranges from 0 to 32, with higher scores indicating a higher stress. A previous study showed that the J‐ZBI_8 has good reliability and validity.^18^ We used a cutoff score of 12/13 based on a previous study, which suggests that caregivers are at risk for depression if they score 13 or more.^24^

#### 2.2.2 Explanatory variable: Primary care experience when family caregivers report as patients

We used the Japanese version of the Primary Care Assessment Tool Short Form (JPCAT‐SF)^25^ to assess primary care experiences. The original JPCAT is an internationally established Patient‐Reported Experience Measure (PREM) in primary care, which was developed based on the Primary Care Assessment Tool created by the Johns Hopkins Primary Care Policy Center.^26^, ^27^ To improve the ease of response, the JPCAT‐SF consists of fewer items than the original JPCAT. It first assesses whether the subject has a usual source of care by asking “Is there a doctor that you usually go if you are sick or need advice about your health?”, and, if they do, it measures patient‐reported experience in primary care, with 13 items. This 13‐item scale is composed of six multi‐item subscales, representing primary care attributes, each of which has a range of 0–100 points. The subscales comprise first contact, longitudinality, coordination, comprehensiveness (services available), comprehensiveness (services provided), and community orientation. The brief description of each subscale is as follows.

*First contact* is closely related to “access to care” and is measured in the JPCAT‐SF by two items related to out‐of‐hours care in primary care. *Longitudinality* is evaluated by two items regarding whether the person feels that their physician recognizes them as a whole person. *Coordination* is measured by three items, asking about the experiences of referral to a specialist. If respondents answer that they have never seen a specialist or are unsure if they have been referred, 50 points (the middle number of the possible scores) are given. *Comprehensiveness (services available)* is measured by two items regarding whether the person feels they can receive care for abuse and advanced care planning as needed. *Comprehensiveness (services provided)* is measured by two items regarding whether the person received advice on self‐medication and health literacy. *Community orientation* is measured by two items regarding whether the person feels that their physician is interested in the needs and health issues of the community.

The JPCAT‐SF score is the average of the six subscale scores and reflects the overall primary care experience, with higher scores indicating better experience. A previous study showed that the JPCAT‐SF has good reliability and validity.^25^

#### 2.2.3 Covariates

Covariates were selected based on clinical priority and a literature review to identify factors that may confound the association between primary care experience that family caregivers reported as patient and their stress related to caregiving. We included covariates for age, gender, relationships with care recipients, self‐rated health, educational attainment, annual household income, average daily caregiving time, instrumental support from relatives or acquaintances, whether the family caregiver's physician and care recipient's physician are the same, and family caregivers' experience with care recipients' providers. Average daily caregiving time was determined using a question based on the Comprehensive Survey of Living Conditions (CSLC) questionnaire by the Japanese government.^16^ According to the study using the Japanese version of the Zarit Caregiver Burden Interview, from which the J‐ZBI_8 was derived, the average daily caregiving time strongly reflects the care recipient's activities of daily living deficits and was the factor strongly affecting caregiver stress (burden).^28^ To assess instrumental support from relatives or acquaintances, we used the following item from previous research: “I have relatives or acquaintances who help me with errands”^29^ because the extent of our questionnaire was limited. Participants chose from responses ranging from 1 (Disagree) to 5 (Agree). Whether the family caregiver's physician and care recipient's physician are the same was asked in the next question following the JPCAT‐SF questionnaire: “Please tell us whether the physician is the same one who regularly provides medical care to the person being cared for.” Family caregivers' experience with care recipients' providers was measured using The Japanese version of the Caregivers' Experience Instrument (J‐IEXPAC CAREGIVERS).^30^ J‐IEXPAC CAREGIVERS is a scale that quantitatively evaluates the care of care recipients' providers, such as physicians, nurses, and care managers, from the family caregiver's perspective (ranging from 12 to 60).

### 2.3 Statistical analysis

We calculated descriptive statistics for family caregivers' characteristics. A modified Poisson regression model was used to determine whether the JPCAT‐SF score was positively associated with caregivers' stress related to caregiving. We analyzed the unadjusted association between the JPCAT‐SF score and the outcome by calculating the crude prevalence ratio in bivariate regression.

In multivariate modified Poisson regression, the following variables were included as possible confounders: age, gender, relationships with care recipients, self‐rated health, educational attainment, annual household income, average daily caregiving time, instrumental support from relatives or acquaintances, whether the family caregiver's physician and the care recipient's physician are the same, and family caregivers' experience with care recipients' providers. Variables except for JPCAT‐SF score, age, instrumental support, and family caregivers' experience with care recipients' providers were divided into multiple categorical variables. Instrumental support was treated as a continuous variable, based on Norman's argument for the validity of treating ordinal variables as continuous variables.^31^

Additionally, we used the same model as in the primary analysis to perform analyses of the association between the scores for each of the JPCAT‐SF subscales and caregiver stress. In the exploratory analyses, comparisons were repeated without Bonferroni correction.^32^ We accounted for missing data in independent and dependent variables by using multiple imputations with a fully conditional specification. Statistical analyses were conducted using SPSS Statistics version 29 (IBM Corp).

## 3 RESULTS

### 3.1 Participants' characteristics

**FIGURE 1:**
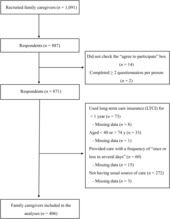
Flowchart of participants.

Of the 1091 family caregivers recruited, 887 (81.3%) responded to the questionnaire, with 406 ultimately included in the analysis. Figure 1 shows the flow chart of the study participants. Table 1 shows the distribution of the 406 family caregivers' characteristics. The mean (standard deviation, SD) age of the family caregivers was 62.9 (6.9) years, and the majority were women (76.6%). About half the family caregivers (52.5%) provided ≥2–3 h of care, per day. The mean (SD) JPCAT‐SF total score was 42.1 (16.3) out of 100 points. Among the subscales of the JPCAT‐SF, first contact scored the lowest (27.2), whereas coordination scored the highest (53.4). The proportion of family caregivers who had higher caregiver stress was 48.8%.

**TABLE 1:** Family caregivers' characteristics (*N* = 406).

Characteristic	Total	Caregiver stress*		
	(*N* = 406)	Lower	Higher	Missing
		(*N* = 201)	(*N* = 198)	(*N* = 7)
Age (years), mean (SD)	62.9 (6.9)	63.6 (6.7)	61.7 (7.1)	
Gender, *N* (%)				
Men	95 (23.4)	48 (23.9)	44 (22.2)	3
Women	311 (76.6)	153 (76.1)	154 (77.8)	4
Relationships with care recipients, *N* (%)				
Spouse	84 (20.7)	51 (25.4)	31 (15.7)	2
Child	229 (56.4)	113 (56.2)	113 (57.1)	3
Child‐in‐law	79 (19.5)	31 (15.4)	47 (23.7)	1
Other	14 (3.4)	6 (3.0)	7 (3.5)	1
Self‐rated health, *N* (%)				
Poor	28 (6.9)	13 (6.6)	15 (7.6)	0
Not very good	78 (19.2)	32 (16.2)	44 (22.3)	2
Good	242 (59.6)	121 (61.1)	117 (59.4)	4
Very good	54 (13.3)	32 (16.2)	21 (10.7)	1
Missing	4			
Education, *N* (%)				
Did not complete high school	18 (4.4)	12 (6.0)	6 (3.0)	0
High school	189 (46.6)	91 (45.5)	96 (48.5)	2
Career college, junior college, or higher professional school	117 (28.8)	56 (28.0)	59 (29.8)	2
College or graduate school	81 (20.0)	41 (20.5)	37 (18.7)	3
Missing	1			
Annual household income (million JPY), *N* (%)				
<2.50 (about USD 20,000)	145 (35.7)	72 (35.8)	70 (35.5)	3
2.50–4.99	167 (41.1)	85 (42.3)	78 (39.6)	4
5.00–7.99	57 (14.0)	26 (12.9)	31 (15.7)	0
≥8.00	36 (8.9)	18 (9.0)	18 (9.1)	0
Missing	1			
Caregiving time per day, *N* (%)				
Almost all‐day	65 (16.0)	24 (11.9)	39 (19.7)	2
Half day	80 (19.7)	31 (15.4)	48 (24.2)	1
2–3 h	68 (16.7)	25 (12.4)	41 (20.7)	2
Lend a hand when needed	193 (47.5)	121 (60.2)	70 (35.4)	2
Instrumental support from relatives or acquaintances**, mean (SD)	3.5 (1.4)	3.8 (1.3)	3.4 (1.4)	
Whether the family caregiver's physician and care recipient's physician are the same				
Same	149 (37.1)	74 (37.6)	74 (37.4)	1
Different	253 (62.9)	123 (62.4)	124 (62.6)	6
Missing	4			
Family caregivers' experience with care recipients' providers***, mean (SD)	41.6 (8.1)	41.9 (8.2)	41.4 (7.9)	
JPCAT‐SF, mean (SD)				
Total score	42.1 (16.3)	44.2 (15.5)	40.0 (16.9)	
First contact	27.2 (25.0)	27.6 (24.4)	26.5 (26.0)	
Longitudinality	48.2 (26.2)	49.6 (25.2)	45.7 (26.9)	
Coordination	53.4 (28.5)	56.5 (28.5)	50.8 (28.2)	
Comprehensiveness (services available)	45.9 (27.8)	50.5 (27.3)	42.0 (28.0)	
Comprehensiveness (services provided)	31.1 (30.4)	33.0 (31.4)	29.9 (30.0)	
Community orientation	47.1 (18.0)	48.1 (19.7)	45.3 (20.8)	

Abbreviations: JPCAT‐SF, Japanese version of the Primary Care Assessment Tool Short Form; J‐ZBI_8, Japanese short version of the Zarit Caregiver Burden Interview; SD, standard deviation.

* Measured using the Japanese short version of the Zarit Caregiver Burden Interview (J‐ZBI_8): lower, the J‐ZBI_8 score < 13; higher, the J‐ZBI_8 score ≥ 13.

** “I have relatives or acquaintances who help me with errands” with responses ranging from 1 (Disagree) to 5 (Agree).

*** Measured using the Japanese version of the Caregivers' Experience Instrument (J‐IEXPAC CAREGIVERS), with total scores ranging from 12 to 60.

### 3.2 Associations of the JPCAT‐SF scores with caregiver stress

In bivariate (unadjusted) models, the higher the JPCAT‐SF score, the more significantly lower was the caregiver stress (lower stress = 0 vs. higher stress = 1; crude prevalence ratio [PR] per 1 SD increase in JPCAT‐SF score = 0.87; 95% CI 0.79–0.96).

Table 2 shows the results of multivariate modified Poisson regression analyses of the association of the JPCAT‐SF score and six subscale scores with caregiver stress. After adjusting for possible confounders, the JPCAT‐SF score was still significantly associated with caregiver stress (adjusted PR per 1 SD increase = 0.89; 95% CI 0.80–0.98). Among the subscales, longitudinality and comprehensiveness (services available) were associated with caregiver stress.

**TABLE 2:** Associations of the JPCAT‐SF scores with caregiver stress (*N* = 406).

JPCAT‐SF	Adjusted PR (95% CI)*	*p*‐Value
Total score	0.89 (0.80–0.98)	0.021
Subscale scores		
First contact	0.98 (0.88–1.08)	0.718
Longitudinality	0.90 (0.81–1.00)	0.047
Coordination	0.93 (0.84–1.02)	0.106
Comprehensiveness (services available)	0.87 (0.79–0.96)	0.003
Comprehensiveness (services provided)	0.96 (0.87–1.06)	0.440
Community orientation.	0.96 (0.86–1.06)	0.401

*Note*: Each score was included separately in the model. All models were adjusted for age, gender, relationships with care recipients, self‐rated health, educational attainment, annual household income, average daily caregiving time, instrumental support from relatives or acquaintances, whether the family caregiver's physician and care recipient's physician are the same, and family caregivers' experience with care recipients' providers.

Abbreviations: CI, confidence interval; JPCAT‐SF, Japanese version of the Primary Care Assessment Tool Short Form; PR, prevalence ratio.

* Per 1 SD increase.

## 4 DISCUSSION

Our results showed that better primary care experiences reported by family caregivers as patients were associated with lower stress related to caregiving. When analyzed by primary care attributes, higher scores on longitudinality and comprehensiveness (services available) were significantly associated with lower stress. These findings indicate that high‐quality primary care, particularly longitudinality and comprehensiveness (services available), may contribute to reducing caregiver stress.

Our findings were similar to those of previous surveys and qualitative studies showing that good quality primary care plays an important role in supporting family caregivers who are patients. A survey in Germany found that family caregivers valued the following types of care and support provided by their family physicians (general practitioners): early recognition by the physician that a family caregiver is responsible for someone's care; and advice by the physician about local support and assistance services in providing care.^10^ A study of family physicians and primary care teams in Canada also found that the continuity of primary care promotes relationships and trust between primary care providers and family caregivers, as caregivers who are patients usually return to the clinic.^33^ Family caregivers may be positively influenced in their caregiving experience by person‐centered, high‐quality primary care.

Among the quality indicators of person‐centeredness measured by the JPCAT‐SF, our results showed that longitudinality and comprehensiveness (services available) can contribute to reducing caregivers' stress related to caregiving. We could make several hypotheses about the mechanisms of how these primary care attributes influence caregiver stress. In the JPCAT‐SF, longitudinality is evaluated by whether the person feels that their physician recognizes them as a whole person.^25^ Physicians who provide holistic care with longitudinal responsibility recognize that the person in front of them provides care for their loved ones, and may naturally engage in conversations regarding caregiving. Such conversations may provide emotional support and, as a result, reduce caregivers' stress related to caregiving. Similarly, in the JPCAT‐SF, comprehensiveness (services available) is measured by whether the person feels they can receive care for abuse and advanced care planning as needed.^25^ Although the items in this subscale do not include direct wording regarding caregiving, family caregivers who experienced better comprehensiveness (services available) may have felt that they could receive advice and support regarding caregiving from their physicians. This could contribute to reducing the caregiver stress.

However, contrary to our expectations, coordination was not significantly associated with family caregivers' stress related to caregiving in this study. One review of family physicians' views on their role in supporting family caregivers recommended collaborative integrated primary care models as the most feasible for supporting caregivers, including coordinating care and assisting caregivers to navigate health and community systems.^34^ Starfield, one of the developers of the original Primary Care Assessment Tool (PCAT), claimed that the essence of coordination is “the availability of information about prior, and existing problems and services, and the recognition of that information as it bears on needs for current care.”^35^ However, the PCAT coordination subscale focuses only on service coordination between primary care providers and specialists.^27^ This is also the same for the JPCAT‐SF. Nearly 50% of our study participants answered that they had never been referred to a specialist or were unsure if they had been referred to one, resulting in skewed score distribution of 50 points, which probably influenced our results. There is no doubt that the role of primary care physicians, working simultaneously with other services, is essential in supporting family caregivers.^34^

This study has several limitations. First, among the multiple factors that influence caregiver stress, the variables used in this study were limited. Notably, we did not include basic information on the care recipient in this study out of respect for the ethical challenge we faced when attempting to obtain direct care recipient's consent at collection. However, we included caregiving time, which reflects care recipient’ deficit in activities of daily living, and also included participants' demographic characteristics and self‐rated health, which have often been adjusted in studies using Person (Patient)‐Reported Experience Measures (PREMs). Second, there could be selection bias because the target age of the participants was limited to 40–74 years. According to the Comprehensive Survey of Living Conditions questionnaire by the Japanese government in 2019, the proportion of caregivers in this age group accounts for approximately 70%.^36^ Third, since the setting of this study was limited to one prefecture in Japan, it is necessary to pay attention to its applicability to other settings. However, the impact on the study results could be limited because the distribution of JPCAT‐SF scores in this study was similar to that in a representative sample of Japanese adults.^37^ Further, the distribution of J‐ZBI_8 lower/higher groups was similar to that in a study conducted in other parts of Japan.^38^ Fourth, the study design was cross‐sectional, and thus a causal relationship could not be definitively established. These limitations thus call for further investigation.

Despite these limitations, to the best of our knowledge, this pilot study is the first to reveal an association between family caregivers' primary care experience and their stress, using the internationally established measure as a tool for assessing the quality of primary care. Expectations are pinned on primary care providers as agents who care about the needs, wishes, and stresses of family caregivers.^4^ Findings from this study, showing that focusing attention on the individual as a whole person and building provider‐patient relationships that make it easier to seek consultations when needed were associated with lower stress related to caregiving, may encourage primary care providers and stakeholders.

## 5 CONCLUSIONS

We found that better primary care experiences when family caregivers report as patients were associated with lower caregiver stress. In particular, longitudinality and comprehensiveness (services available) may contribute to reducing the caregiver stress. Despite several limitations, our findings suggest that high‐quality primary care may provide positive experiences for family caregivers in the context of caregiver stress.

## CONFLICT OF INTEREST STATEMENT

The authors declare no conflict of interest in connection with this article.

## ETHICS STATEMENTS

The survey was approved by the Ethics Committee of the Faculty of Medicine, University of Tsukuba (approval no. 1518‐2). This committee grants approval to studies based on the provisions of the Declaration of Helsinki and Japanese ethical guidelines (Ethical Guidelines for Medical and Health Research Involving Human Subjects).

## PATIENT CONSENT STATEMENT

All participants were volunteers and checked the box on the questionnaire indicating their intention to participate.

## CLINICAL TRIAL REGISTRATION

None.
